# Steering Mast Cells or Their Mediators as a Prospective Novel Therapeutic Approach for the Treatment of Hematological Malignancies

**DOI:** 10.3389/fonc.2021.731323

**Published:** 2021-09-24

**Authors:** Deeksha Mehtani, Niti Puri

**Affiliations:** Cellular and Molecular Immunology Lab, School of Life Sciences, Jawaharlal Nehru University, New Delhi, India

**Keywords:** hematological malignancy, mast cells, lymphoid neoplasms, lymphoma, leukemia, blood cancer, cancer therapeutics, myeloma

## Abstract

Tumor cells require signaling and close interaction with their microenvironment for their survival and proliferation. In the recent years, Mast cells have earned a greater importance for their presence and role in cancers. It is known that mast cells are attracted towards tumor microenvironment by secreted soluble chemotactic factors. Mast cells seem to exert a pro-tumorigenic role in hematological malignancies with a few exceptions where they showed anti-cancerous role. This dual role of mast cells in tumor growth and survival may be dependent on the intrinsic characteristics of the particular tumor, differences in tumor microenvironment according to tumor type, and the interactions and heterogeneity of mediators released by mast cells in the tumor microenvironment. In many studies, Mast cells and their mediators have been shown to affect tumor survival and growth, prognosis, inflammation, tumor vascularization and angiogenesis. Modulating mast cell accumulation, viability, activity and mediator release patterns may thus be important in controlling these malignancies. In this review, we emphasize on the role of mast cells in lymphoid malignancies and discuss strategies for targeting and steering mast cells or their mediators as a potential therapeutic approach for the treatment of these malignancies.

## 1. Introduction

Cancer is a complicated disease and a leading cause of mortalities, the world over. Hematological malignancies are the most common and frequently occurring cancers in children and the elderly (1, 2). Development of these malignancies is characterized by a crucial transition of hematopoietic cells of a particular lineage to cancerous cells with altered and abnormal cellular proliferation. One such condition that is most commonly seen in elderly people is clonal hematopoiesis, a premalignant disorder characterized by aberrant proliferation of clonally-derived hematopoietic stem cells carrying somatic mutations in leukemia-related genes. Aside from age advancement, this phenomenon is more common in solid or lymphoid tumors and is linked to genotoxic stress (3). Leukemia refers to the clonal expansion of abnormal leukocyte cells in the bone marrow (BM), which leads to the elevated levels of affected cells in the blood circulation. While lymphoma or lymphoid malignancies show elevated numbers of B or T lymphocytes which are present as tumor in the lymphatic tissue (1). Due to their complexity, the hematologic malignancies become challenging to manage.

Cancer pathogenesis involves multiple interactions between neoplastic cells and their microenvironment resulting in maintenance and progression or rejection of growing tumor. For hematological malignancies, BM or secondary lymphoid organs form the cancer microenvironment which is composed of stromal cells, fibroblasts, immune cells and vascular endothelial cells (4). Dynamic signaling by soluble mediators or cell-cell interactions between leukemia or lymphoma cells and immune cells in the tumor or surrounding microenvironment strongly determines the malignant progression or eradication of tumor. Presence of immune cells also plays a greater role in tumor development or elimination. CD8^+^ T cells and NK cells eradicate immunogenic cancer cells which leave the variants of cells that are non-immunogenic, making it difficult for the immune system to recognize them (5). Many studies have associated the presence of Mast cells (MCs) and release of various mediators with the remodeling of tumor microenvironment.

MCs are innate immune granulocytes, derived from bone marrow, that migrate to peripheral tissues to mature and reside in mucosal layers and near blood vessels remaining close to the external environment so as to respond quickly to an invasion by a pathogen or allergen (6). Years ago, Paul Ehrlich discovered MCs and found them to be present in close proximity to a tumor (7). MCs have a wide range of surface receptors like FcέRI, histamine receptors, c-KIT receptor, Pattern recognition receptors (PRRs) which on activation make them capable of releasing diverse set of mediators in response to various stimuli (8). In tumor microenvironment, MCs release molecules like Vascular endothelial growth factor (VEGF), heparin, tryptase, Fibroblast growth factor (FGF-2) which can initiate tumor angiogenesis and molecules like Matrix metalloproteinases (MMP-9 and MMP-2) which can enable tumor niche remodeling, migration and invasiveness collectively leading to cancer progression (9). Whereas secreted molecules like histamine, IL-4, IL-8, Tumor necrosis factor (TNF-α) contribute in inhibiting tumor cell survival or growth and inducing apoptosis (9). MC functions are extremely context-dependent and cross-talk between tumor cells-MCs and other tumor-associated immune cells are likely to play a role in determining whether a tumor will be eliminated or progressed.

The existing anti-cancerous treatments focus on targeting the mechanisms behind the abnormally proliferating cells. Therefore, an unmet need in cancer research is to understand the cancer microenvironment and the interplay between tumor cells and the immune cells like MCs which on activation release a diverse variety of mediators having capacity to modulate the tumor microenvironment in favor of or for rejection of cancer. In this review, we emphasize on the role of MCs in hematological malignancies and discuss the strategies to target and steer MCs or their mediators as a potential therapeutic approach for these malignancies.

### 1.1 Dual Role of MCs in Hematological Malignancies

The contribution of immune and inflammatory cells, such as MCs, is well known in the control, progression and invasion of malignant cells. Here we discuss the studies highlighting the correlation between the amount of tumor-infiltrating MCs and the extent of tumor aggressiveness and propagation, implying a significant role of MCs in various hematological malignancies. The characteristics of the studies reviewed have been compiled in **Tables 1** and **2**.

**Table 1:** Characteristics of clinical studies included in this review mentioning the stage of hematological malignancies and number of mast cells.

Type of hematological malignancy	Stage of hematological malignancy	Mast cell number	Role played by MCs	Year of publication	Reference
Hodgkin’s Lymphoma	subtypes of CHL (NSCHL and non-NSCHL)	Higher number of MCs in IL-13 positive HRS cell group	Pro-tumorigenic	2016	(10)
	Stage I-IIA Stage IIB-IV	The disease-free survival rate and overall survival rate were both lower in patients with a higher MC count		2002	(11)
	NS subtype, non NS subtype, MxC subtype	CD30L is expressed by MCs, and there is no difference in MCs numbers between groups.		2001	(12)
	Stage I-II Stage III-IV, Histological type-NS subtype MxC subtype	MCs were more in nodular sclerosis (NS) than mixed cellularity (MxC) subtype		2015	(13)
Splenic Marginal Zone Lymphoma	Grouped according to IIL score (Low risk, intermediate risk, high risk)	MCs express CD40L	Pro-tumorigenic	2014	(14)
Primary Cutaneous Lymphoma (PCL)	Mycosis fungoides (MF) Stage IA-IB Stage IIA-IIB	MC number and density higher in early stages of MF IA and IB	Pro-tumorigenic	2016	(15)
	For MF, FMF, SS Stage IA-IB Stage IIA-IIB Stage IIIIB Stage IVA	MC number and degranulation increase in CTCL and CBCL		2012	(16)
B cell Lymphoma (BCL)	DLBCL (Diffuse large B cell lymphoma) Stage III-IV	Not mentioned	Pro-tumorigenic	2016	(17)
	Stages Not mentioned Samples from DLBCL, FL, Mantle cell lymphoma, marginal zone B cell lymphoma			2011	(18)
T cell Lymphoma (TCL)	Stages not mentioned, samples from BCL, TCL	MC numbers were higher in TCL than BCL	Pro-tumorigenic	2001	(19)
	Stage not mentioned. Samples from AITL (Angioimmunoblastic T cell lymphoma), PCL	MC numbers were higher in AITL than PCL		2010	(20)
Leukemia	CML Phases mentioned Chronic Phase, Accelerated Phase and Blast Phase (ALLT, AMLT)	MC numbers were highest in AMLT (Acute myeloid leukemia transformation stage)	Pro-tumorigenic	2019	(21)
Multiple Myeloma (MM)	Stage I, II, III	MC density was higher in advanced stages of MM	Pro-tumorigenic	2013	(22)
	Stage I, II, III	The diseased state had a higher MC density than the healthy control.		2015	(23)
	Active MM (Stage I, II, III)	The diseased state had a higher MC density than the healthy control group, and it was even higher in advanced stages of MM.		2016	(24)

**Table 2:** Experimental setup of the studies included in this review.

Type of hematological malignancy	Experimental setup	Demonstrated role played by mast cells	Year of publication	Reference
Hodgkin’s Lymphoma	*In vitro* (human HL cell lines L428, HDLM2,KMH2; human leukemia cell line HL60; mouse BMMC, SPMCs *In vivo* (NOD/SCID mice)	Pro-tumorigenic	2012	(25)
	Patient sample		2016	(10)
	Patient sample		2002	(11)
	Patient sample, *In vitro* (HMC-1, KU812)		2001	(12)
	Patient Sample		2015	(13)
	*In vitro* (HL cell lines CO, DEV, HDLM-2,KMH2,L540), umbilical cord derived CBMC		2003	(26)
Splenic Marginal Zone Lymphoma	Patient Sample, *In vitro*	Pro-tumorigenic	2014	(14)
Primary Cutaneous Lymphoma (PCL)	Archival tissue samples of patients	Pro-tumorigenic	2016	(15)
	Patient samples *In vitro* (Mac2B, MyLa, SeAx, BJAB^30^, Jurkat, HMC-1, EL4)*, In vivo* (C57BL/6 *Kit^W-sh/W-sh^* mice and transgenic mast cell–deficient *Mcpt5-Cre^+^/iDTR^+^* mice)		2012	(16)
B cell Lymphoma (BCL)	Patient samples	Pro-tumorigenic	2016	(17)
	Patient samples *In vitro* (mouse BCL cell line A20) *In vivo* (BALB/c)		2011	(18)
T cell Lymphoma (TCL)	Patient samples	Pro-tumorigenic	2001	(19)
	Patient samples *In vitro* (BJAB, LAD2, ADMEC)		2010	(20)
	*In vitro* (murine TCL cell line EL4), RBL-2H3 mast cell line		2019	(27)
	*In vitro* (murine TCL cell line YAC-1), RBL-2H3 mast cell line	Anti-cancerous		
Leukemia	*In vitro* (murine leukemia cell line L1210), RBL-2H3 mast cell line	No effect	2019	(27)
	Patient sample	Pro-tumorigenic	2019	(21)
Multiple myeloma	Patient sample	Pro-tumorigenic	2013	(22)
	Patient sample	Not shown	2020	(28)
	Patient sample	Pro-tumorigenic	2015	(23)
	*In vivo* (syngeneic mouse MM model using IgA-producing plasmocytoma MOPC-315 cells, MOPC-104E and J588 plasmocytomas originating from Balb/c)	Not shown	2015	(29)
	Patient sample	Pro-tumorigenic	2016	(24)
Melanoma	*In vivo* (C57BL/6, B6.129S6-*Tnf^tm1Gkl^*/J, B6.129S2-*Il6^tm1Kopf^*/J, B6.129P2-*Ccl3^tm1Unc^*/J, and B6.Cg-*Kit^W-sh^*/HNihrJaeBsmJ (*Kit^W-sh/W-sh^*) *In vitro* (B16.F10 cells, BMMC)	Anti-cancerous	2010	(30)
Colon cancer	Patient sample *In vivo* (azoxymethane induced CAC model, *Cysltr1^−/−^* C57BL/6)	Anti-cancerous	2016	(31)
Colorectal cancer	Patient sample	Anti-cancerous	2018	(32)

BMMC, Bone marrow-derived mast cells; SPMC, Spleen-derived mast cells; CBMC, cord blood-derived mast cells; CAC, colitis-associated colorectal cancer.

#### 1.1.1 The Role of Mast Cells in Lymphomas

MCs have been documented to be involved in shaping the microenvironment in lymphomas, mostly by increasing the microvessel density, increasing angiogenesis and fibrosis thus leading to rogue advancement of lymphomas. Hodgkin’s lymphoma (HL), derived from mature B cells, is characterized by tumor cells, Hodgkin’s and Reed-Sternberg (HRS) cells in a smoldering inflammatory microenvironment (33). Abundance in tryptase positive MCs has been predominantly associated with inflammation and poor prognosis in patients with HL. Infiltration of TGF-β producing MCs in HL’s subtype-nodular sclerosis has been associated with the invasion of neoplastic cells, the development of fibrosis and progression of HL by the promotion of angiogenesis (10, 25). MCs were shown to promote HL cell growth in SCID mice *in vivo* (25). In HL, MCs have also been reported to interact directly with tumor cells *via* CD30-CD30L, causing HRS cells to become activated and proliferate could be important for HL pathogenesis (10–12). Indirect interactions between tumor cells and MCs caused by soluble factors produced by HRS cells, such as IL-9, IL-13, CCL5/RANTES are important for MC infiltration and proliferation (10, 11, 13, 26). MCs are, therefore, involved in shaping the HL microenvironment in terms of angiogenesis and fibrosis leading to advancement of tumor cells towards invasion and nodular progression.

Splenic marginal zone lymphoma (SMZL), is characterized by indolent neoplastic B cells that infiltrate the spleen and sometimes the BM (34). MCs are directly recruited by neoplasm cells in the tumor microenvironment and support the stromal cell proliferation, angiogenesis, extracellular matrix (ECM) remodeling in this B cell malignancy. Also, stromal cells highly express CD40 which recruits MCs expressing CD40 ligand, lead to the release of IL-6 with other pro-inflammatory cytokines, thereby activating B cells, increasing the survival and proliferation of neoplastic cells and contributing to pathobiology of SMZL progression (14).

B cell lymphoma (BCL) accounts for 90% of lymphoid neoplasms worldwide. The most common and aggressive form of non-Hodgkin lymphoma that occurs within the lymph nodes, but can be present anywhere in the body outside the lymphoid system, is diffuse large B cell lymphoma (DLBCL). Marinaccio et al. speculated that a decrease in MC density would result in reduced inflammatory signals and increased pro-angiogenic signals by regulatory T cells (T_regs_) (17). Whereas, an increase in MC density can enhance inflammation and suppress the functions of T_regs_ thereby allowing the differentiation and expansion of T_h_17 lymphocytes leading to angiogenesis (17). Feng et al. considered IL-9 to be a key driver of tumor growth by which T_regs_ recruited and activated MCs to mediate immune suppression in the tumor region (18). The interplay of infiltrating T_regs_ and MCs in the tumor microenvironment therefore promotes the formation of tumor vessels, maintains the growth and metastasis of tumor in BCL.

Primary cutaneous lymphoma (PCL) is a non-Hodgkin’s lymphoma also known as lympho-proliferative neoplasm of clonal B cell or T cell lymphoma largely associated with the skin (35). Studies have shown that there is an increased number of MCs present in both the cutaneous B cell lymphoma (CBCL) and cutaneous T cell lymphoma (CTCL) in peripheral rims of skin (15). In CTCL presence of MCs is correlated with reduced survival and increased malignancy in patients with progressive disease or advanced disease stage in Folliculotropic mycosis fungoides and Se´zary syndrome compared to stable patients or early disease stage in Mycosis fungoides (MF) (16). MCs are not only present in advanced stages of CTCL and CBCL, but have also extensively degranulated, which is much more noticeable in progressive form of these neoplasms, whereas more non-degranulated form of MCs are present in MF (15). *In vitro* study has shown that the supernatant of MCs obtained by treatment with calcium ionophore can induce the production of cytokines such as IL-17, IL-6 from tumor cells and increase the proliferation of primary CTCL cells (16). For the first time, Rabenhorst et al. have used a connective tissue-MCs depleted mouse model to demonstrate the delay in development of PCL thus highlighting the crucial role of MCs in controlling the tumor progression (16). Rabenhorst et al. also demonstrated that adding MC supernatant increased the proliferation of Sezary and CTCL cell lines whereas MC supernatant had no effect on the proliferation of SeAx and Mac2B CTCL cell lines when MC degranulation was inhibited by cromolyn (16). Therefore MCs play a pro-tumorigenic role in PCL, which is critical in the advanced stages of the disease and can be linked to disease severity.

T cell lymphomas (TCLs) account for the rare group of non-Hodgkin’s lymphoma group due to their low prevalence. Angioimmunoblastic TCL (AITL) is the uncommon aggressive subtype of the mature peripheral TCL that involves lymph node (36) and dysregulation of T cell immune response (37). The presence of micro-vessels with high endothelial venules is a prominent feature of AITL. There is strong correlation between MCs and number of blood vessels in TCL cases studied by Fukushima et al. Their study suggests that MCs are responsible for the angiogenesis and progression of AITL (19). Neoplastic follicular T_h_ cells in AITL have been shown to produce CXCL-13, which is responsible for the accumulation of MCs strongly expressing IL-6 in AITL speculated to foster a pro-inflammatory microenvironment and deregulated angiogenesis (20). In addition to studies documenting the pro-tumorigenic role of MCs in TCL, YAC-1 T cell lymphoma cells in direct contact with MCs or tumor cell supernatant when added to MCs was shown to induce degranulation of MCs (27). Interestingly, after co-treatment with histamine receptor antagonists and MC mediators, it was discovered that histamine receptors H2 and H4 are involved in inhibition of YAC-1 cell growth whereas histamine receptors H1, H2 and H4 are involved in enhancement of EL4 T cell lymphoma cell growth and overall regulation of β catenin pathway (27). Furthermore, Rabenhorst et al. demonstrated that MCs are crucial for the progression of EL4 TCL tumors *in vivo* using an inducible mast cell deficiency mouse model *Mcpt5-Cre/iDTR* and *Kit* mutant mice. Increased proliferation of EL4 cells and release of pro-inflammatory cytokines on *in vitro* treatment with MC or BMMC supernatant was also observed (16). MCs exhibited a pro-tumorigenic role on the EL4 TCL cell line, but they exhibited an anti-cancerous role on the YAC-1 TCL cell line, implying that MCs may exist as picket cells in the lymphoma microenvironment and play a critical role in the suppression or advancement of tumorigenesis, depending on tumor characteristics and histamine receptor profile present on neoplastic cells (27).

#### 1.1.2 The Role of Mast Cells in Leukemia

As discussed above there is a pro-inflammatory and pro-cancerous role of MCs in various lymphomas with an exception in a T cell lymphoma. It was also demonstrated that MCs showed no effect on the proliferation of L1210 cell line which is a murine lymphocytic leukemia cell line *in vitro* (27). Similar to lymphomas, tryptase positive MCs were found to be abundant in the BM of chronic myeloid leukemia (CML) patients and increased MCs number in different stages of CML conformed to increased microvessel density and the advancement of CML to AML, which is the blast phase or the advanced stage (21). Interestingly, MCs count in acute lymphoblastic leukemia transformation (ALLT) stage was comparable to that of the healthy control group, implying that MCs control angiogenesis in the early stages of tumor development, whereas tumor cells drive growth and angiogenesis in later stages, and growth becomes MC-independent (21).

#### 1.1.3 The Role of Mast Cells in Myeloma

Multiple myeloma (MM) is the malignancy of neoplastic plasma cells infiltrating the bone marrow. Similar to lymphomas and leukemias, patients with MM have an infiltration of tryptase positive MCs in their BM, associated with increased neovascularization and angiogenesis (22). Cytokines such as IL-6, VEGF, TNF-α, B cell activating factor, and receptor activator of NF-kB ligand are elevated in MM (28). IL-6 is required for the survival and proliferation of normal immature B cells in the BM, and it has been identified as the key growth and survival factor for myeloma cells (28). Raised IL-6 levels can be caused by both myeloma precursor cells and the presence of MCs (23, 29). Increased MC density has also been linked to increased angiogenesis factors found in BM of MM patients (24). Therefore, MC can either directly or indirectly contribute to the progression of MM.

### 1.2 Mast Cells and Conventional Cancer Therapy

Conventional cancer therapy involves chemotherapy and radiotherapy which are principally used to kill the rapidly dividing tumor cells. According to Soule et al, MCs are resistant to cytotoxicity after radiation, and radiation has no effect on Kit and FcεRI receptor expression. MCs degranulation is inhibited transiently and recovers within 24 hours after irradiation in human MCs, and MCs remain responsive to TLR-mediated signalling and produce cytokines (38). Westbury et al. observed a post-irradiation increase in the number of MCs (39). Radiation can also cause MC degranulation and the release of mediators like tryptase, as well as increase vascular permeability, which can lead to tissue injuries and fibrosis (40, 41). MCs may even be responsible for resistance to anti-PD1 therapy, which is linked to lower expression of HLA class1 in tumor cells, resulting in tumor escape from cytotoxic T cells (42). In prostate cancer, MCs can induce docetaxel resistance by phosphorylating p38 and radio-resistance by phosphorylating ATM, resulting in tumor cell survival and proliferation (43). Similarly, in inflammatory breast and pancreatic cancer, MCs have been implicated in tumor cell resistance to therapy and can also reduce the effect of anti-angiogenic therapy (44–46). As a result, MCs have emerged as important candidate cells in the tumor microenvironment to be targeted for therapies.

### 1.3 Various Strategies to Target Mast Cells as a Potential Therapeutic Approach

**Figure 1:**
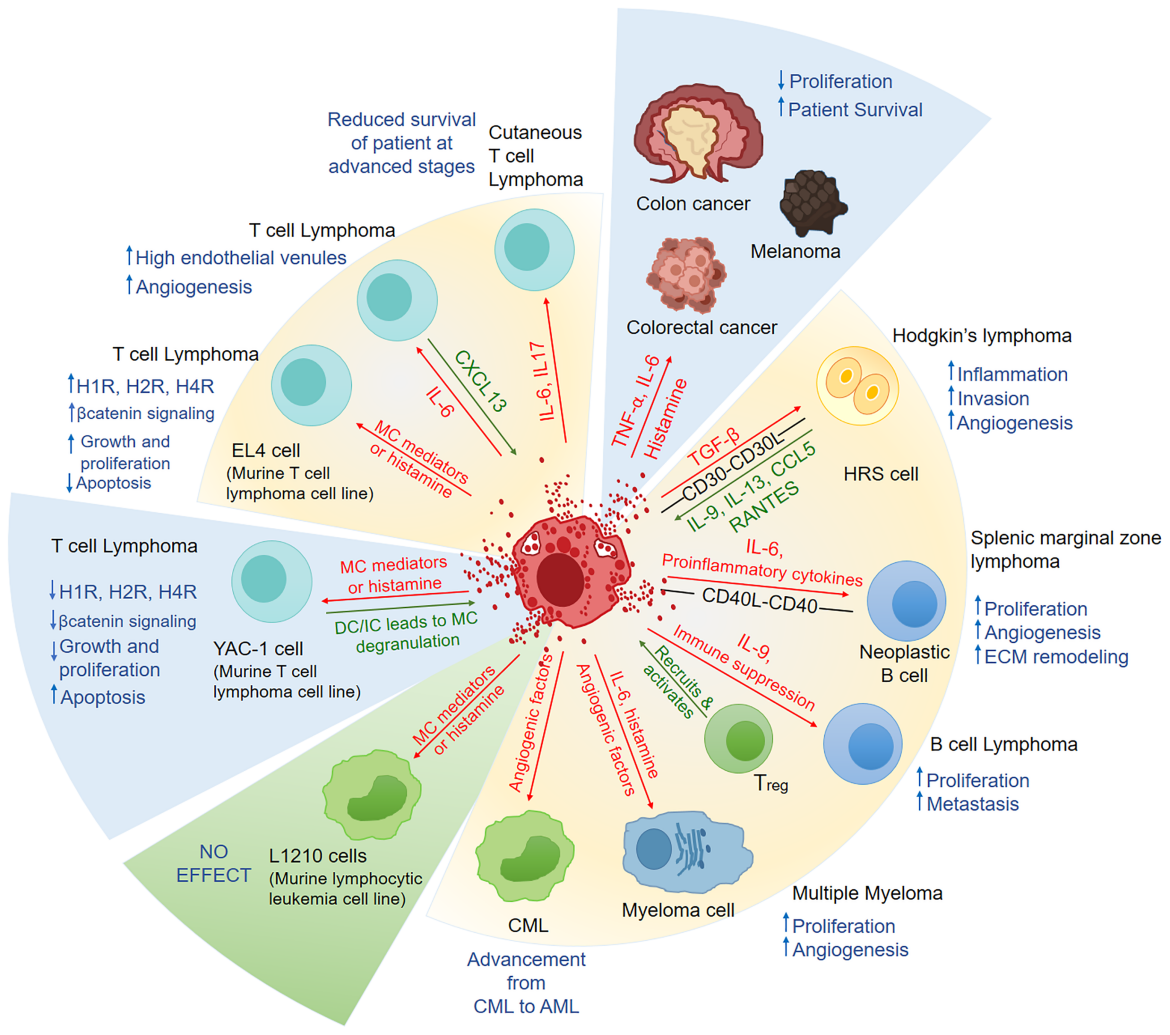
A summary of the factors released by mast cells and their physiological response in hematological malignancies and solid cancers. In Hodgkin’s lymphoma, splenic marginal zone lymphoma, B cell lymphoma, CML, T cell lymphoma, and myeloma MCs have been shown to play pro-tumorigenic role. In an *in-vitro* study, MCs had an anti-tumorigenic effect in T cell lymphoma but had no effect in murine lymphocytic leukemia. In some solid cancers, like colon cancer, melanoma and colorectal cancers, MCs have been shown to play anti-cancerous role. The factors released by MCs are represented by red arrows in this figure, while the factors/chemokines released by leukemia/lymphoma cells are represented by green arrows. Blue upwards arrow represents the increase and blue downwards arrow represents the decrease, as a result of the effect of MC on the malignancy. In the pie representation, blue slice represents the anti-cancerous role, green slice represents No effect and yellow represents pro-tumorigenic role played by MCs. MC, Mast cell; HRS, Hodgkin’s and Reed-Sternberg cells; ECM,extracellular matrix; Treg, Regulatory T cells; CML, Chronic myeloid leukemia; AML, acute myeloid leukemia; H1R:, H1 Histamine Receptor; H2R, H2 Histamine Receptor; H4R, H4 Histamine Receptor; DC, Direct contact; IC, Indirect contact means MCs treated with tumor cell supernatant only.

As discussed in section 1.1, accumulation of MCs and their precise role in almost all hematological malignancies were evidently correlated with detrimental effects, poor prognosis, angiogenesis, tumor aggressiveness and metastasis as summarized in **Figure 1**. Increase in number of MCs in early stages is correlated as an angiogenic switch which triggers the tumor towards the malignant advancement (21). Myeloid derived suppressor cells (MDSCs), Tregs, NK cells have been extensively studied for their tumor mediated immunosuppressive activity and ability to impair immunotherapy response (47). The interplay between MCs and MDSCs can be speculated as a builder for inflammatory tumor microenvironment as MCs secrete CCL2 which can recruit MDSCs and subsequent IL-17 secretion recruits Tregs producing IL-9 which is required for maintenance of MCs contributing to the immunosuppressive microenvironment (48, 49) making MCs as an important target for a responsive immunotherapy.

MCs are an excellent candidate for targeted immunotherapy in the microenvironment of hematological malignancies because of their increased number, selective release of variety of mediators upon activation, and interaction with other cells. In this section we will discuss various strategies that could potentially help target MCs in the hematological tumor microenvironment.

#### 1.3.1 Strategies to Target Mast Cell Number

c-KIT receptor is critical for the survival and development of MCs as MC depletion is shown in mouse models with c-KIT mutation (50). The targeting of c-KIT receptor is therefore one such strategy that may help to reduce the number of MCs in the tumor microenvironment of hematological malignancies. c-KIT is a tyrosine kinase receptor and can be targeted by numerous tyrosine kinase inhibitors used as anti-cancer drugs such as imatinib, sunitinib, sofrafenib, which bind and inhibit Bcr-abl fusion protein tyrosine kinase and are currently being used in CML (51), gastro-intestinal cancers and in thymic carcinoma (52). Other United States Food and Drug Administration (FDA) approved anti-cancer drugs that are studied to target c-kit include Amuvatinib, which has been clinically tested for lymphoma and small cell lung carcinomas, Axitinib, was clinically tested for advanced renal cell carcinoma, Cabozantinib, for prostate cancers and Dasatinib, for Chronic myeloid leukemia (52). A biologic inhibitor of c-KIT, KT0158, which is a humanized monoclonal antibody, has been shown to decrease MC degranulation and reduce MC numbers in a preclinical study (53).

In addition to c-KIT inhibitors, interestingly Fluvastatin, a statin drug used in the treatment of hypercholesterolemia not only suppresses IgE signaling in MCs but also induces apoptosis by inhibiting stem cell factor (SCF) induced survival signals in primary and in c-KIT mutated MCs (54). Consequently, the strategy to target the number of MCs would serve as an anti-inflammatory approach with a potential to suppress neo-vascularization, leading to a considerable delay in the tumor growth prior to the initiation of angiogenic switch. This strategy could prove to be a rational and effective additional therapeutic strategy for lymphoid neoplasms negatively affected by MCs.

#### 1.3.2 Mast Cell Stabilization

MCs release pre-formed mediators present within their granules and newly formed lipid mediators instantly upon activation and other newly synthesized mediators are released 3-12 hours later. T cell lymphoma cells have been shown to activate MCs to a similar extent as an allergen and show similar release of pre-stored mediators (27). These mediators are further responsible for the inflammation and angiogenic progression in lymphoid neoplasms or the rejection of tumors as seen in some solid cancers like breast cancer. Therefore, if it is not possible to eliminate MCs, we can think about stabilization of MCs. MC Stabilization is a method of preventing the release of histamine and other mediators involved in rogue actions by impeding degranulation.

Bortezomib, an NF-Kβ inhibitor used to treat MM and other malignant hematological disorders, has been found to be minimally cytotoxic to MCs but can block MCs release, preventing fibrosis and vascularization in HL tumors *in vivo*. As a result, Bortezomib may be an interesting molecule to be used to kill malignant cells while simultaneously inactivating MCs in tumors, whereas Mizuno et al. claim that monotherapy may not work because the effect is transient (25).

Since histamine receptors are involved in responses to MC mediators, the use of MC antagonists may be helpful in reversing their response. Many MC stabilizers are now known to inhibit MC activation, such as H1 Histamine receptor antagonist Ketotifen, which is used to treat asthma and has been shown to suppress fibrosis (55). Azelastine, H1 receptor antagonist is a potent anti-inflammatory molecule that also inhibits release of histamine, tryptase and IL-6 from MCs (56). To inhibit histamine receptors in TCL cell lines, Pyrilamine as an antagonist for H1 Histamine receptor, Ranitidine as an antagonist for H2 histamine receptor, and JNJ7777120 as an antagonist for H4 receptor have been used (27). Some Tyrosine kinase inhibitors which are anti-cancerous agents such as Nilotinib, Sunitinib, Ibrutinib have been shown to have anti-histamine properties and can work as MC stabilizers (57–59). Not only chemical sources, but some natural sources of MC stabilizers such as flavonoids like luteolin, amentoflavone, bilobetin, quercetin (60, 61), phenols like curcumin (62) and alkaloids such as theanine present in green tea (63) have been shown to have antihistamine and MC stabilizing properties. It is therefore essential to identify and study how these stabilizers can be incorporated as add on therapeutic agents for the treatment of hematological malignancies.

#### 1.3.3 MC Pre-Formed Mediators That Can Be Targeted or Incorporated in Cancer Therapies

As discussed in the section 1.1, MCs identified in haematological malignancies are tryptase-positive and are capable of causing neo-vascularization by secreting tryptase and chymase, which are potent angiogenic factors (64). Gabexate mesylate (GM), Nafamostat mesylate (NM) are tryptase inhibitors. NM is 100 times more potent than GM which is used in the treatment of acute pancreatitis and has been tested on several human cancer cell lines and Tranilast is used in the treatment of bronchial asthma and has a potential anti-tumor activity (65). Therefore, these molecules may be incorporated into treatments. As a result, targeting tryptase released by MC in the tumor microenvironment will serve as an anti-angiogenic strategy.

Although MCs have been shown to be involved in tumor progression, heparin, a MC mediator has been studied for its anti-cancerous role in many solid cancers (66). Heparin has been shown to attenuate metastasis in experimental cancer models possibly by inhibiting blood coagulation, inhibiting cancer cell–platelet and –endothelial interactions by selectin inhibition (67). It is therefore essential to study the role of heparin in hematological malignancies and thus strategies for including this molecule in cancer therapy.

Histamine is an important molecule that is pre-stored in MC granules and is the first molecule released when MCs are activated. Histamine has also been shown to be involved in cell proliferation, tumor development and embryonic development (68). The anti-cancerous properties of histamine in TCL have also been reported. Studies showed that histamine inhibits proliferation, decreases the survival and induces apoptosis in YAC-1 TCL cell line *in vitro* (27). Histamine dihydrochloride, a NOX2 inhibitor, targets MDSCs and improves immune-mediated clearance of neoplastic cells, thereby improving the immunotherapy efficacy of PD1 checkpoint blockade (69). Because of its receptor expression on immune cells as well as on tumor cells, histamine becomes an important molecule that can be strategically included in anti-cancer therapy and can have a detrimental effect on tumor depending on the construction of tumor microenvironment, cell types present and the histamine receptor expression profile.

#### 1.3.4 Anti-Tumor Immune Responses of MCs

Apart from the pro-tumorogenic role, MCs have been shown to have an anti-tumor role in some solid cancers such as melanoma, colon cancer, and colorectal cancer where MC presence in tumor is associated with improved patient survival (30–32), and also in a haematological neoplasm *in vitro* (27).

The tumor microenvironment contains sufficient chemokines and alternate molecules that can activate MCs. Chemotherapy and radiotherapy, as well as injury, cause dead and dying cells to release molecules known as alarmins or Danger-associated molecular patterns (DAMPs), which can activate MCs *via* TLRs and other receptors (70, 71). In hematological malignancies, alarmins such as IL-33 and Hsp-70 are commonly released, and cytokines such as IL-1 have been found to activate MCs and cause the release of IL-6 and TNF-α, while chymase from MCs has been shown to degrade IL-33 and Hsp-70 due to their ability to cause inflammation (70, 72, 73). Depending on the activation molecule and receptor activated on MC, the anti-tumor mechanism involves recruiting immune effector cells such as NK cells and cytotoxic lymphocytes, which can eventually lead to tumor cell clearance or the development of anti-tumor immunity. Virus activated MCs have been shown to degranulate and produce type I and type III interferons, CXCL8, CXCR1, and TNF, which can recruit and activate IFN-γ producing NK cells and NKT cells, allowing them to carry out cytotoxic actions which eliminate transformed cells (74–76). Interferon-stimulated chemokines have been found in tumors (32), therefore interferon-induced MC activation and NK cells recruitment may play a role in anti-tumor immunity.

Histamine, an important mediator released by MCs, is involved in the conservation of cytotoxicity receptors (NKp46, NKG2D) on NK cells in AML that are likely to be inhibited by phagocytes, and histamine prevents this inhibition by targeting H2 receptors on phagocytes (77). Histamine was used as a protector for cytotoxic lymphocytes and NK cells from phagocytes in a clinical study on AML where IL-2 was to be given as immunotherapy, which delayed relapse in patients and significantly improved the therapy (78). When activated, MCs also produce lipid mediators such as prostaglandins, leukotrienes, which are also involved in the recruitment of cytotoxic lymphocytes. As demonstrated in colorectal cancers that Leukotriene B4 derived from MC is in charge of recruiting and homing CD8+ T cells to the tumor site in order to generate anti-tumor immunity (79). As a result, MCs contribute chemokines, granule-associated and *de novo* synthesized mediators in tumor microenvironment, which may be important for immune regulation and anti-tumor responses.

To improve the recruitment of effector cells by MCs, innate immune activators such as TLR targeting immune-therapeutic molecules could be used. TLR-2 activated MCs have been shown to recruit NK cells and T cells in a melanoma model (30), whereas TLR-3 receptor activation on MCs increases T cell recruitment and regulates its functions (80). Therefore, TLR agonists can be combined with molecules that inhibit the release of pro-tumorigenic factors by MCs (as discussed in previous section), resulting in an effective anti-tumor response.

## 2 Conclusion and Future Perspectives

The presence of MCs has been increasingly recognized in human cancers. Pathological studies of MCs in human tissues have revealed contradictory results, explaining both a positive and a negative correlation between the number of MCs and prognosis in various cancers. The role of MCs in hematological malignancies has been the focus of this mini review as the role of MCs in tumorigenesis is of increasing interest following the use of anti-tumor agents that affect tumor growth by inhibiting factors known to be crucial to MC function.

MCs are found to be beneficial for the tumor growth in hematological neoplasms, as discussed in this review, with the exception of T cell lymphomas, where an *in vitro* study showed that MCs were detrimental to tumor cell growth. The role of MCs may be influenced by the stage of tumor development at which they infiltrate, their interaction with other cells, and the tumor microenvironment. MCs have been discussed to trigger the angiogenic switch in the tumors that helps to nourish the tumor and later on makes the tumor growth independent of MCs. MCs pro-tumorigenic effects are primarily mediated by angiogenic molecules secretion, tissue remodelling, tumor cell proliferation augmentation, and immunosuppression. Because MCs have the ability to influence cellular recruitment, proliferation, and functioning, they might be critical regulators of tumor microenvironment and, as a result tumor growth.

MCs can be activated by various molecules present in tumor microenvironment and are capable of secreting different mediators in response to different triggers. Sometimes they may only secrete cytokines without any release of pre-formed mediators. Also there is heterogeneity even in pre-stored mediators and their secretion is controlled by different secretion machinery (81). In such a scenario, it may be possible to specifically target and block secretion of pro-angiogenic factors and allow secretion of mediators which may have anti-tumor activities. Also it becomes important to understand the interactions of MCs with cancer stem cells or under hypoxic condition which is a hallmark of tumor microenvironment.

All of this tends to suggest that research into the role of MCs in hematological malignancies could have direct clinical implications in the use of targeted therapies, and that it should be investigated further using histopathological and appropriate multifaceted biological models. Some molecules or drugs that have been used for other purposes but can interfere with SCF-cKIT signaling could be repurposed, or some lysomotrophic drugs that cause granule permeabilization and eventually apoptosis of MCs could be geared at selectively limiting the number of MCs which may significantly reduce the inflammatory background that inevitably leads to aggravation and invasion of tumor cells. In addition, we discussed some therapeutic strategies which are available or can be considered to inhibit the viability, accumulation and interfere with activation of MCs and their mediator release to enhance the anti-tumor response. In the context of hematological malignancies, one potential therapeutic mechanism could be to activate MCs using TLR-activators after chemotherapy/radiotherapy to boost the innate immune response and effector cell recruitment. TLR-activators may be used in conjunction with histamine. Antihistamines or MC stabilizers could be used in cases where there is increase in pro-tumorigenic or angiogenic molecules, for example histamine regulates myeloma cell growth. Interplay between MCs and Tregs is responsible for angiogenesis in BCL, therefore could be inhibited by reducing the numbers of MCs and Tregs. Thus, a decision about how to incorporate MCs in immunotherapy can be made based on the malignancy’s microenvironment, chemokines, alarmins released, and the extent and type of histamine receptors expressed.

## Author Contributions

DM contributed to literature search, data curation and wrote the first draft of article. NP contributed to conception and design of the study, project administration and finalized the manuscript. All authors contributed to the article and approved the submitted version.

## Funding

This study was supported by research grants from Department of Science and Technology (DST) - Science & Engineering Board (SERB) Govt. of India (CRG/2019/003651) to NP. DM received senior research fellowship (2020-7400/SCR-BMS) from ICMR.

## Conflict of Interest

The authors declare that the research was conducted in the absence of any commercial or financial relationships that could be construed as a potential conflict of interest.

## Publisher’s Note

All claims expressed in this article are solely those of the authors and do not necessarily represent those of their affiliated organizations, or those of the publisher, the editors and the reviewers. Any product that may be evaluated in this article, or claim that may be made by its manufacturer, is not guaranteed or endorsed by the publisher.

## References

[1] Bispo JAB Pinheiro PS Kobetz EK. Epidemiology and Etiology of Leukemia and Lymphoma. Cold Spring Harbor Perspect Med (2020) 10:a034819. doi: 10.1101/cshperspect.a034819 PMC7263093 31727680

[2] Satyanarayana L Asthana S Labani SP. Childhood Cancer Incidence in India: A Review of Population-Based Cancer Registries. Indian Pediatr (2014) 51:218–20. doi: 10.1007/s13312-014-0377-0 24736911

[3] Park SJ Bejar R. Clonal Hematopoiesis in Cancer. Exp Hematol (2020) 83:105–12. doi: 10.1016/j.exphem.2020.02.001 PMC7103485 32044376

[4] Hopken UE Rehm A. Targeting the Tumor Microenvironment of Leukemia and Lymphoma. Trends Cancer (2019) 5:351–64. doi: 10.1016/j.trecan.2019.05.001 31208697

[5] Gonzalez H Hagerling C Werb Z. Roles of the Immune System in Cancer: From Tumor Initiation to Metastatic Progression. Genes Dev (2018) 32:1267–84. doi: 10.1101/gad.314617.118 PMC6169832 30275043

[6] Elieh Ali Komi D Wohrl S Bielory L. Mast Cell Biology at Molecular Level: A Comprehensive Review. Clin Rev Allergy Immunol (2020) 58:342–65. doi: 10.1007/s12016-019-08769-2 31828527

[7] Ghably J Saleh H Vyas H Peiris E Misra N Krishnaswamy G. Paul Ehrlich’s Mastzellen: A Historical Perspective of Relevant Developments in Mast Cell Biology. Methods Mol Biol (2015) 1220:3–10. doi: 10.1007/978-1-4939-1568-2_1 25388241

[8] Kim HS Kawakami Y Kasakura K Kawakami T. Recent Advances in Mast Cell Activation and Regulation. F1000Research (2020) 9:F1000 Faculty Rev-196. doi: 10.12688/f1000research.22037.1 PMC7096214 32226609

[9] Ribatti D Crivellato E. Mast Cells, Angiogenesis, and Tumour Growth. Biochim Biophys Acta (2012) 1822:2–8. doi: 10.1016/j.bbadis.2010.11.010 21130163

[10] Nakayama S Yokote T Hiraoka N Nishiwaki U Hanafusa T Nishimura Y. Role of Mast Cells in Fibrosis of Classical Hodgkin Lymphoma. Int J Immunopathol Pharmacol (2016) 29:603–11. doi: 10.1177/0394632016644447 PMC5806829 27095287

[11] Molin D Edstrom A Glimelius I Glimelius B Nilsson G Sundstrom C. Mast Cell Infiltration Correlates With Poor Prognosis in Hodgkin’s Lymphoma. Br J Haematol (2002) 119:122–4. doi: 10.1046/j.1365-2141.2002.03768.x 12358914

[12] Molin D Fischer M Xiang Z Larsson U Harvima I Venge P. Mast Cells Express Functional CD30 Ligand and Are the Predominant CD30L-Positive Cells in Hodgkin’s Disease. Br J Haematol (2001) 114:616–23. doi: 10.1046/j.1365-2141.2001.02977.x 11552987

[13] Andersen MD Kamper P Nielsen PS Bendix K Riber-Hansen R Steiniche T. Tumour-Associated Mast Cells in Classical Hodgkin’s Lymphoma: Correlation With Histological Subtype, Other Tumour-Infiltrating Inflammatory Cell Subsets and Outcome. Eur J Haematol (2016) 96:252–9. doi: 10.1111/ejh.12583 25963595

[14] Franco G Guarnotta C Frossi B Piccaluga PP Boveri E Gulino A. Bone Marrow Stroma CD40 Expression Correlates With Inflammatory Mast Cell Infiltration and Disease Progression in Splenic Marginal Zone Lymphoma. Blood (2014) 123:1836–49. doi: 10.1182/blood-2013-04-497271 24452203

[15] Eder J Rogojanu R Jerney W Erhart F Dohnal A Kitzwogerer M. Mast Cells Are Abundant in Primary Cutaneous T-Cell Lymphomas: Results From a Computer-Aided Quantitative Immunohistological Study. PloS One (2016) 11:e0163661. doi: 10.1371/journal.pone.0163661 27893746 PMC5125565

[16] Rabenhorst A Schlaak M Heukamp LC Forster A Theurich S von Bergwelt-Baildon M. Mast Cells Play a Protumorigenic Role in Primary Cutaneous Lymphoma. Blood (2012) 120:2042–54. doi: 10.1182/blood-2012-03-415638 22837530

[17] Marinaccio C Ingravallo G Gaudio F Perrone T Ruggieri S Opinto G. T Cells, Mast Cells and Microvascular Density in Diffuse Large B Cell Lymphoma. Clin Exp Med (2016) 16:301–6. doi: 10.1007/s10238-015-0354-5 25957593

[18] Feng LL Gao JM Li PP Wang X. IL-9 Contributes to Immunosuppression Mediated by Regulatory T Cells and Mast Cells in B-Cell Non-Hodgkin’s Lymphoma. J Clin Immunol (2011) 31:1084–94. doi: 10.1007/s10875-011-9584-9 21898141

[19] Fukushima N Satoh T Sano M Tokunaga O. Angiogenesis and Mast Cells in non-Hodgkin’s Lymphoma: A Strong Correlation in Angioimmunoblastic T-Cell Lymphoma. Leukemia lymphoma (2001) 42:709–20. doi: 10.3109/10428190109099333 11697501

[20] Tripodo C Gri G Piccaluga PP Frossi B Guarnotta C Piconese S. Mast Cells and Th17 Cells Contribute to the Lymphoma-Associated Pro-Inflammatory Microenvironment of Angioimmunoblastic T-Cell Lymphoma. Am J Pathol (2010) 177:792–802. doi: 10.2353/ajpath.2010.091286 20595635 PMC2913370

[21] Xu P Zhang C Wang Y Wu H Cheng S Fan X. Increased Number of Mast Cells in the Bone Marrow of Chronic Myeloid Leukemia may Herald the Pending Myeloid Transformation—The Mast Cell Is an Indicator of Myeloid Transformation. Trans Cancer Res (2019) 8:2121–9. doi: 10.21037/tcr.2019.09.29 PMC8797750 35116962

[22] Pappa CA Tsirakis G Roussou P Xekalou A Goulidaki N Konsolas I. Positive Correlation Between Bone Marrow Mast Cell Density and ISS Prognostic Index in Patients With Multiple Myeloma. Leukemia Res (2013) 37:1628–31. doi: 10.1016/j.leukres.2013.09.012 24183234

[23] Devetzoglou M Vyzoukaki R Kokonozaki M Xekalou A Pappa CA Papadopoulou A. High Density of Tryptase-Positive Mast Cells in Patients With Multiple Myeloma: Correlation With Parameters of Disease Activity. Tumour biology: J Int Soc Oncodevelopmental Biol Med (2015) 36:8491–7. doi: 10.1007/s13277-015-3586-9 26026586

[24] Vyzoukaki R Tsirakis G Pappa CA Androulakis N Kokonozaki M Tzardi M. Correlation of Mast Cell Density With Angiogenic Cytokines in Patients With Active Multiple Myeloma. Clin Ther (2016) 38:297–301. doi: 10.1016/j.clinthera.2015.11.022 26740291

[25] Mizuno H Nakayama T Miyata Y Saito S Nishiwaki S Nakao N. Mast Cells Promote the Growth of Hodgkin’s Lymphoma Cell Tumor by Modifying the Tumor Microenvironment That Can Be Perturbed by Bortezomib. Leukemia (2012) 26:2269–76. doi: 10.1038/leu.2012.81 22430634

[26] Fischer M Juremalm M Olsson N Backlin C Sundstrom C Nilsson K. Expression of CCL5/RANTES by Hodgkin and Reed-Sternberg Cells and Its Possible Role in the Recruitment of Mast Cells Into Lymphomatous Tissue. Int J Cancer (2003) 107:197–201. doi: 10.1002/ijc.11370 12949794

[27] Paudel S Mehtani D Puri N. Mast Cells may Differentially Regulate Growth of Lymphoid Neoplasms by Opposite Modulation of Histamine Receptors. Front Oncol (2019) 9:1280. doi: 10.3389/fonc.2019.01280 31824856 PMC6881378

[28] Jasrotia S Gupta R Sharma A Halder A Kumar L. Cytokine Profile in Multiple Myeloma. Cytokine (2020) 136:155271. doi: 10.1016/j.cyto.2020.155271 32916474

[29] Matthes T Manfroi B Zeller A Dunand-Sauthier I Bogen B Huard B. Autocrine Amplification of Immature Myeloid Cells by IL-6 in Multiple Myeloma-Infiltrated Bone Marrow. Leukemia (2015) 29:1882–90. doi: 10.1038/leu.2015.145 26159051

[30] Oldford SA Haidl ID Howatt MA Leiva CA Johnston B Marshall JS. A Critical Role for Mast Cells and Mast Cell-Derived IL-6 in TLR2-Mediated Inhibition of Tumor Growth. J Immunol (2010) 185:7067–76. doi: 10.4049/jimmunol.1001137 21041732

[31] Mehdawi L Osman J Topi G Sjolander A. High Tumor Mast Cell Density Is Associated With Longer Survival of Colon Cancer Patients. Acta Oncol (2016) 55:1434–42. doi: 10.1080/0284186X.2016.1198493 27355473

[32] Mao Y Feng Q Zheng P Yang L Zhu D Chang W. Low Tumor Infiltrating Mast Cell Density Confers Prognostic Benefit and Reflects Immunoactivation in Colorectal Cancer. Int J Cancer (2018) 143:2271–80. doi: 10.1002/ijc.31613 29873076

[33] Canioni D Deau-Fischer B Taupin P Ribrag V Delarue R Bosq J. Prognostic Significance of New Immunohistochemical Markers in Refractory Classical Hodgkin Lymphoma: A Study of 59 Cases. PloS One (2009) 4:e6341. doi: 10.1371/journal.pone.0006341 19623262 PMC2710003

[34] Piris MA Onaindia A Mollejo M. Splenic Marginal Zone Lymphoma. Best Pract Res Clin Haematol (2017) 30:56–64. doi: 10.1016/j.beha.2016.09.005 28288718

[35] Willemze R Cerroni L Kempf W Berti E Facchetti F Swerdlow SH. The 2018 Update of the WHO-EORTC Classification for Primary Cutaneous Lymphomas. Blood (2019) 133:1703–14. doi: 10.1182/blood-2018-11-881268 PMC6473500 30635287

[36] Lunning MA Vose JM. Angioimmunoblastic T-Cell Lymphoma: The Many-Faced Lymphoma. Blood (2017) 129:1095–102. doi: 10.1182/blood-2016-09-692541 28115369

[37] Iannitto E Ferreri AJ Minardi V Tripodo C Kreipe HH. Angioimmunoblastic T-Cell Lymphoma. Crit Rev Oncol/Hematol (2008) 68:264–71. doi: 10.1016/j.critrevonc.2008.06.012 18684638

[38] Soule BP Brown JM Kushnir-Sukhov NM Simone NL Mitchell JB Metcalfe DD. Effects of Gamma Radiation on Fcepsilonri and TLR-Mediated Mast Cell Activation. J Immunol (2007) 179:3276–86. doi: 10.4049/jimmunol.179.5.3276 17709544

[39] Westbury CB Freeman A Rashid M Pearson A Yarnold JR Short SC. Changes in Mast Cell Number and Stem Cell Factor Expression in Human Skin After Radiotherapy for Breast Cancer. Radiotherapy Oncol: J Eur Soc Ther Radiol Oncol (2014) 111:206–11. doi: 10.1016/j.radonc.2014.02.020 24746564

[40] Albrecht M Muller K Kohn FM Meineke V Mayerhofer A. Ionizing Radiation Induces Degranulation of Human Mast Cells and Release of Tryptase. Int J Radiat Biol (2007) 83:535–41. doi: 10.1080/09553000701444657 17613126

[41] Park KR Monsky WL Lee CG Song CH Kim DH Jain RK. Mast Cells Contribute to Radiation-Induced Vascular Hyperpermeability. Radiat Res (2016) 185:182–9. doi: 10.1667/RR14190.1 PMC4761451 26771172

[42] Somasundaram R Connelly T Choi R Choi H Samarkina A Li L. Tumor-Infiltrating Mast Cells Are Associated With Resistance to Anti-PD-1 Therapy. Nat Commun (2021) 12:346. doi: 10.1038/s41467-020-20600-7 33436641 PMC7804257

[43] Xie H Li C Dang Q Chang LS Li L. Infiltrating Mast Cells Increase Prostate Cancer Chemotherapy and Radiotherapy Resistances *via* Modulation of P38/P53/P21 and ATM Signals. Oncotarget (2016) 7:1341–53. doi: 10.18632/oncotarget.6372 PMC4811464 26625310

[44] Reddy SM Reuben A Barua S Jiang H Zhang S Wang L. Poor Response to Neoadjuvant Chemotherapy Correlates With Mast Cell Infiltration in Inflammatory Breast Cancer. Cancer Immunol Res (2019) 7:1025–35. doi: 10.1158/2326-6066.CIR-18-0619 PMC7053657 31043414

[45] Porcelli L Iacobazzi RM Di Fonte R Serrati S Intini A Solimando AG. Cafs and TGF-Beta Signaling Activation by Mast Cells Contribute to Resistance to Gemcitabine/Nabpaclitaxel in Pancreatic Cancer. Cancers (2019) 11:cancers11030330. doi: 10.3390/cancers11030330 PMC6468868 30866547

[46] Wroblewski M Bauer R Cubas Cordova M Udonta F Ben-Batalla I Legler K. Mast Cells Decrease Efficacy of Anti-Angiogenic Therapy by Secreting Matrix-Degrading Granzyme B. Nat Commun (2017) 8:269. doi: 10.1038/s41467-017-00327-8 28814715 PMC5559596

[47] Draghiciu O Lubbers J Nijman HW Daemen T. Myeloid Derived Suppressor Cells-an Overview of Combat Strategies to Increase Immunotherapy Efficacy. Oncoimmunology (2015) 4:e954829. doi: 10.4161/21624011.2014.954829 25949858 PMC4368153

[48] Danelli L Frossi B Pucillo CE. Mast Cell/MDSC a Liaison Immunosuppressive for Tumor Microenvironment. Oncoimmunology (2015) 4:e1001232. doi: 10.1080/2162402X.2014.1001232 26137400 PMC4485753

[49] Jachetti E Cancila V Rigoni A Bongiovanni L Cappetti B Belmonte B. Cross-Talk Between Myeloid-Derived Suppressor Cells and Mast Cells Mediates Tumor-Specific Immunosuppression in Prostate Cancer. Cancer Immunol Res (2018) 6:552–65. doi: 10.1158/2326-6066.CIR-17-0385 29523597

[50] Grimbaldeston MA Chen CC Piliponsky AM Tsai M Tam SY Galli SJ. Mast Cell-Deficient W-Sash C-Kit Mutant Kit W-Sh/W-Sh Mice as a Model for Investigating Mast Cell Biology *In Vivo* . Am J Pathol (2005) 167:835–48. doi: 10.1016/S0002-9440(10)62055-X PMC1698741 16127161

[51] Sacha T. Imatinib in Chronic Myeloid Leukemia: An Overview. Mediterranean J Hematol Infect Dis (2014) 6:e2014007. doi: 10.4084/MJHID.2014.007 PMC3894842 24455116

[52] Abbaspour Babaei M Kamalidehghan B Saleem M Huri HZ Ahmadipour F. Receptor Tyrosine Kinase (C-Kit) Inhibitors: A Potential Therapeutic Target in Cancer Cells. Drug Design Dev Ther (2016) 10:2443–59. doi: 10.2147/DDDT.S89114 PMC4975146 27536065

[53] London CA Gardner HL Rippy S Post G La Perle K Crew L. KTN0158, a Humanized Anti-KIT Monoclonal Antibody, Demonstrates Biologic Activity Against Both Normal and Malignant Canine Mast Cells. Clin Cancer Res: an Off J Am Assoc Cancer Res (2017) 23:2565–74. doi: 10.1158/1078-0432.CCR-16-2152 PMC5418113 27815356

[54] Paez PA Kolawole M Taruselli MT Ajith S Dailey JM Kee SA. Fluvastatin Induces Apoptosis in Primary and Transformed Mast Cells. J Pharmacol Exp Ther (2020) 374:104–12. doi: 10.1124/jpet.119.264234 PMC7306917 32434944

[55] Gallant-Behm CL Hildebrand KA Hart DA. The Mast Cell Stabilizer Ketotifen Prevents Development of Excessive Skin Wound Contraction and Fibrosis in Red Duroc Pigs. Wound Repair Regen: Off Publ Wound Healing Soc [and] Eur Tissue Repair Soc (2008) 16:226–33. doi: 10.1111/j.1524-475X.2008.00363.x 18318808

[56] Ciprandi G Cosentino C Milanese M Tosca MA. Rapid Anti-Inflammatory Action of Azelastine Eyedrops for Ongoing Allergic Reactions. Ann Allergy Asthma Immunol: Off Publ Am Coll Allergy Asthma Immunol (2003) 90:434–8. doi: 10.1016/S1081-1206(10)61829-7 12722967

[57] El-Agamy DS. Anti-Allergic Effects of Nilotinib on Mast Cell-Mediated Anaphylaxis Like Reactions. Eur J Pharmacol (2012) 680:115–21. doi: 10.1016/j.ejphar.2012.01.039 22329898

[58] Yamaki K Yoshino S. Tyrosine Kinase Inhibitor Sunitinib Relieves Systemic and Oral Antigen-Induced Anaphylaxes in Mice. Allergy (2012) 67:114–22. doi: 10.1111/j.1398-9995.2011.02717.x 21933194

[59] Chang BY Huang MM Francesco M Chen J Sokolove J Magadala P. The Bruton Tyrosine Kinase Inhibitor PCI-32765 Ameliorates Autoimmune Arthritis by Inhibition of Multiple Effector Cells. Arthritis Res Ther (2011) 13:R115. doi: 10.1186/ar3400 21752263 PMC3239353

[60] Kim HP Park H Son KH Chang HW Kang SS. Biochemical Pharmacology of Biflavonoids: Implications for Anti-Inflammatory Action. Arch Pharmacal Res (2008) 31:265–73. doi: 10.1007/s12272-001-1151-3 18409037

[61] Weng Z Zhang B Asadi S Sismanopoulos N Butcher A Fu X. Quercetin Is More Effective Than Cromolyn in Blocking Human Mast Cell Cytokine Release and Inhibits Contact Dermatitis and Photosensitivity in Humans. PloS One (2012) 7:e33805. doi: 10.1371/journal.pone.0033805 22470478 PMC3314669

[62] Lee JH Kim JW Ko NY Mun SH Her E Kim BK. Curcumin, a Constituent of Curry, Suppresses Ige-Mediated Allergic Response and Mast Cell Activation at the Level of Syk. J Allergy Clin Immunol (2008) 121:1225–31. doi: 10.1016/j.jaci.2007.12.1160 18394691

[63] Kim NH Jeong HJ Kim HM. Theanine Is a Candidate Amino Acid for Pharmacological Stabilization of Mast Cells. Amino Acids (2012) 42:1609–18. doi: 10.1007/s00726-011-0847-9 21344174

[64] Ribatti D Ranieri G. Tryptase, a Novel Angiogenic Factor Stored in Mast Cell Granules. Exp Cell Res (2015) 332:157–62. doi: 10.1016/j.yexcr.2014.11.014 25478999

[65] Ammendola M Leporini C Marech I Gadaleta CD Scognamillo G Sacco R. Targeting Mast Cells Tryptase in Tumor Microenvironment: A Potential Antiangiogenetic Strategy. BioMed Res Int (2014) 2014:154702. doi: 10.1155/2014/154702 25295247 PMC4177740

[66] Borsig L. Heparin as an Inhibitor of Cancer Progression. Prog Mol Biol Trans Sci (2010) 93:335–49. doi: 10.1016/S1877-1173(10)93014-7 20807651

[67] Niers TM Klerk CP DiNisio M Van Noorden CJ Buller HR Reitsma PH. Mechanisms of Heparin Induced Anti-Cancer Activity in Experimental Cancer Models. Crit Rev Oncol/Hematol (2007) 61:195–207. doi: 10.1016/j.critrevonc.2006.07.007 17074500

[68] Blaya B Nicolau-Galmes F Jangi SM Ortega-Martinez I Alonso-Tejerina E Burgos-Bretones J. Histamine and Histamine Receptor Antagonists in Cancer Biology. Inflamm Allergy Drug Targets (2010) 9:146–57. doi: 10.2174/187152810792231869 20632959

[69] Grauers Wiktorin H Nilsson MS Kiffin R Sander FE Lenox B Rydstrom A. Histamine Targets Myeloid-Derived Suppressor Cells and Improves the Anti-Tumor Efficacy of PD-1/PD-L1 Checkpoint Blockade. Cancer Immunol immunotherapy: CII (2019) 68:163–74. doi: 10.1007/s00262-018-2253-6 PMC6394491 30315349

[70] Wang Y Su H Yan M Zhang L Tang J Li Q. Interleukin-33 Promotes Cell Survival *via* P38 MAPK-Mediated Interleukin-6 Gene Expression and Release in Pediatric AML. Front Immunol (2020) 11:595053. doi: 10.3389/fimmu.2020.595053 33324412 PMC7726021

[71] Ashrafizadeh M Farhood B Eleojo Musa A Taeb S Najafi M. Damage-Associated Molecular Patterns in Tumor Radiotherapy. Int Immunopharmacol (2020) 86:106761. doi: 10.1016/j.intimp.2020.106761 32629409

[72] Roy A Ganesh G Sippola H Bolin S Sawesi O Dagalv A. Mast Cell Chymase Degrades the Alarmins Heat Shock Protein 70, Biglycan, HMGB1, and Interleukin-33 (IL-33) and Limits Danger-Induced Inflammation. J Biol Chem (2014) 289:237–50. doi: 10.1074/jbc.M112.435156 PMC3879547 24257755

[73] Ronnberg E Ghaib A Ceriol C Enoksson M Arock M Safholm J. Divergent Effects of Acute and Prolonged Interleukin 33 Exposure on Mast Cell Ige-Mediated Functions. Front Immunol (2019) 10:1361. doi: 10.3389/fimmu.2019.01361 31275312 PMC6593472

[74] Portales-Cervantes L Haidl ID Lee PW Marshall JS. Virus-Infected Human Mast Cells Enhance Natural Killer Cell Functions. J Innate Immun (2017) 9:94–108. doi: 10.1159/000450576 27806369 PMC6738812

[75] St John AL Rathore AP Yap H Ng ML Metcalfe DD Vasudevan SG. Immune Surveillance by Mast Cells During Dengue Infection Promotes Natural Killer (NK) and NKT-Cell Recruitment and Viral Clearance. Proc Natl Acad Sci USA (2011) 108:9190–5. doi: 10.1073/pnas.1105079108 PMC3107258 21576486

[76] Burke SM Issekutz TB Mohan K Lee PW Shmulevitz M Marshall JS. Human Mast Cell Activation With Virus-Associated Stimuli Leads to the Selective Chemotaxis of Natural Killer Cells by a CXCL8-Dependent Mechanism. Blood (2008) 111:5467–76. doi: 10.1182/blood-2007-10-118547 18424663

[77] Romero AI Thoren FB Brune M Hellstrand K. Nkp46 and NKG2D Receptor Expression in NK Cells With CD56dim and CD56bright Phenotype: Regulation by Histamine and Reactive Oxygen Species. Br J Haematol (2006) 132:91–8. doi: 10.1111/j.1365-2141.2005.05842.x 16371024

[78] Brune M Castaigne S Catalano J Gehlsen K Ho AD Hofmann WK. Improved Leukemia-Free Survival After Postconsolidation Immunotherapy With Histamine Dihydrochloride and Interleukin-2 in Acute Myeloid Leukemia: Results of a Randomized Phase 3 Trial. Blood (2006) 108:88–96. doi: 10.1182/blood-2005-10-4073 16556892

[79] Bodduluri SR Mathis S Maturu P Krishnan E Satpathy SR Chilton PM. Mast Cell-Dependent CD8(+) T-Cell Recruitment Mediates Immune Surveillance of Intestinal Tumors in Apc(Min/+) Mice. Cancer Immunol Res (2018) 6:332–47. doi: 10.1158/2326-6066.CIR-17-0424 PMC9580996 29382671

[80] Orinska Z Bulanova E Budagian V Metz M Maurer M Bulfone-Paus S. TLR3-Induced Activation of Mast Cells Modulates CD8+ T-Cell Recruitment. Blood (2005) 106:978–87. doi: 10.1182/blood-2004-07-2656 15840693

[81] Puri N Roche PA. Mast Cells Possess Distinct Secretory Granule Subsets Whose Exocytosis Is Regulated by Different SNARE Isoforms. Proc Natl Acad Sci USA (2008) 105:2580–5. doi: 10.1073/pnas.0707854105 PMC2268179 18250339

